# Circulatory Responses to Asphyxia Differ if the Asphyxia Occurs *In Utero* or *Ex Utero* in Near-Term Lambs

**DOI:** 10.1371/journal.pone.0112264

**Published:** 2014-11-13

**Authors:** Kristina S. Sobotka, Colin Morley, Tracey Ong, Graeme R. Polglase, James D. S. Aridas, Suzanne L. Miller, Georg M. Schmölzer, Claus Klingenberg, Timothy J. M. Moss, Graham Jenkin, Stuart B. Hooper

**Affiliations:** 1 The Ritchie Centre, Monash University, Melbourne, Australia; 2 Neonatal Research, Royal Women's Hospital, Melbourne, Australia; 3 Department of Obstetrics and Gynaecology, Monash University, Melbourne, Victoria, Australia; 4 Department of Pediatrics, University Hospital of North Norway, Tromsø, Norway; 5 Pediatric Research Group, Faculty of Health Sciences, University of Tromsø, Tromsø, Norway; University of Catania, Italy

## Abstract

**Background:**

A cornerstone of neonatal resuscitation teaching suggests that a rapid vagal-mediated bradycardia is one of the first signs of perinatal compromise. As this understanding is based primarily on fetal studies, we investigated whether the heart rate and blood pressure response to total asphyxia is influenced by whether the animal is *in utero* or *ex utero*.

**Methods:**

Fetal sheep were instrumented at ∼139 days of gestation and then asphyxiated by umbilical cord occlusion until mean arterial blood pressure decreased to ∼20 mmHg. Lambs were either completely submerged in amniotic fluid (*in utero*; n = 8) throughout the asphyxia or were delivered and then remained *ex utero* (*ex utero*; n = 8) throughout the asphyxia. Heart rate and arterial blood pressure were continuously recorded.

**Results:**

Heart rate was higher in *ex utero* lambs than *in utero* lambs. Heart rates in *in utero* lambs rapidly decreased, while heart rates in *ex utero* lambs initially increased following cord occlusion (for ∼1.5 min) before they started to decrease. Mean arterial pressure initially increased then decreased in both groups.

**Conclusions:**

Heart rate response to asphyxia was markedly different depending upon whether the lamb was *in utero* or *ex utero*. This indicates that the cardiovascular responses to perinatal asphyxia are significantly influenced by the newborn's local environment. As such, based solely on heart rate, the stage and severity of a perinatal asphyxic event may not be as accurate as previously assumed.

## Introduction

Our current understanding of the cardiorespiratory responses to birth asphyxia in humans is primarily based on experiments first conducted in the 1960's by Dawes and colleagues [1]. The first sign of compromise is cessation of respiratory efforts, commonly referred to as primary apnea, which is accompanied by a profound bradycardia. This bradycardia is mediated by vagal inputs and is most strongly initiated by both hypoxia and acidosis [2]. During primary apnea, it is taught that the newborn can be stimulated to resume breathing by actions such as drying or slapping of the feet [3]. If cardiorespiratory compromise continues, the newborn may begin irregular gasping before entering “secondary or terminal” apnea when blood pressure also begins to fall. Once in secondary apnea, it is widely considered that a newborn requires intervention, most commonly in the form assisted ventilation [3].

This understanding is based on many different studies, but was first observed in newborn rhesus monkeys that were made asphyxic by placing a water-filled bag over the newborn's head after delivery [4]–[7]. Subsequently, other studies have examined and characterised this response in more detail, mainly using fetal models, with the fetus remaining *in utero* during asphyxia. In a recent study, we observed that the heart rate response to asphyxia, induced by umbilical cord occlusion, appeared to differ from this characteristic bradycardic response if the lambs were delivered and remained *ex utero* during the asphyxia period [8]. As the *ex utero* lambs were intubated and did not have bags of water over their heads, we hypothesised that that the presence of liquid surrounding the face, may influence the physiological response to birth asphyxia. Our aim was to document and report the cardiovascular responses to birth asphyxia in near-term lambs that either remained *in utero* or were *ex utero* during the period of asphyxia.

## Methods

All experimental procedures were approved by the relevant Monash University Animal Ethics Committee in accordance with the National Health and Medical Research Council (Australia) Australian code of practice for the care and use of animals for scientific purposes (7^th^ Edition, 2004). Pregnant ewes at 139±2 d gestation (term 147 d) were anesthetised and the fetus exposed for instrumentation via a hysterotomy; catheters were implanted into the jugular vein and carotid or femoral artery.

Lambs were then asphyxiated by total occlusion of the umbilical cord. The first group of lambs (n = 8) remained *in utero*, completely submerged in amniotic fluid, throughout the asphyxia period. The second group of lambs (n = 8) were intubated with a clamped cuffed endotracheal tube (4.5mm) to prevent breathing, delivered and remained *ex utero* throughout the period of asphyxia. For this study we analysed data on the circulatory response for the first 10 minutes after occlusion of the cord. Heart rate and arterial blood pressure were continuously recorded (DTX Plus Transducer; Becton Dickinson, Singapore) from prior to delivery until the end of the experiment. Arterial blood gases were collected at the end of asphyxia.

### Analytical methods

Heart rate (HR) and mean arterial pressure (BP) were averaged over 5 s epochs every 30 s 3 min before and for the 10 min study period after umbilical cord occlusion. The carotid arterial pressure waveform was also analysed for end diastolic pressure (BP_ED_; an indicator of downstream vascular resistance) and pulse amplitude (BP_amp_; an indicator of stroke volume). Maximum rate of blood pressure increase during systole (max dP/dt; an indicator of cardiac contractility) was calculated by converting arterial pressure into its first derivative with respect to time.

### Statistical methods

Data were analysed using 2-way repeated measures ANOVA with group (*in utero* vs. *ex utero*) and time as factors. Post hoc comparisons between groups and time points were performed using the Holm-Sidak test. Data are presented as mean ± SEM unless otherwise stated.

## Results

Although the duration of asphyxia was similar between groups at ∼11 min (Table 1) and the target BP for terminating the asphyxia was the same, the BP at end asphyxia was higher in the *ex utero* lambs compared to *in utero* lambs (22.3±4.7 and 17.8±3.6 mmHg respectively). Similarly, at the end of the asphyxia period, PaCO_2_ levels were higher and the pH tended to be lower in *in utero* lambs, compared to *ex utero* lambs (Table 1).

**Table 1:** Lamb arterial blood gases and physiological parameters at the end of asphyxia.

	*in utero*	*ex utero*
Asphyxia duration (min)	11.1±1.4	11.2±1.3
BP (mmHg)	17.8±3.6*	22.3±4.7
pH	6.2±1.9	6.9±0.04
PaCO_2_ (mmHg)	161.3±36.7*	100.8±27.0
PaO_2_ (mmHg)	3.4±1.0	4.9±5.3
SaO_2_ (%)	2.0±0.7*	9.0±6.8
Lactate (mmol/L)	10.1±2.2	8.3±3.3
BE (mmol/L)	−12.2±4.0	−17.0±3.0

BP, arterial blood pressure; PaCO_2_, partial pressure of carbon dioxide; PaO_2_, partial pressure of oxygen; SaO_2_, oxygen saturation; BE, base excess. Data are mean ± SD. * p<0.05 *in utero* vs *ex utero*.

**Figure 1:**
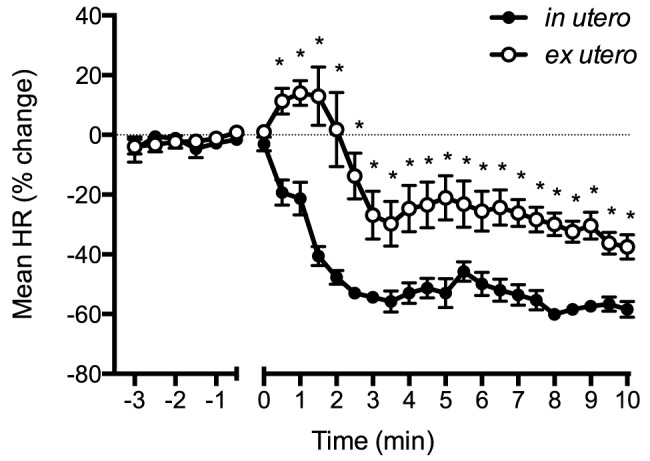
Heart rate (HR), expressed as a percentage change from before cord clamping, measured in in *in utero* (•) or *ex utero* (○) lambs before and after umbilical cord occlusion (designated as time 0). Data are mean ± SEM. * p<0.05 *in utero* vs *ex utero*.

**Figure 2:**
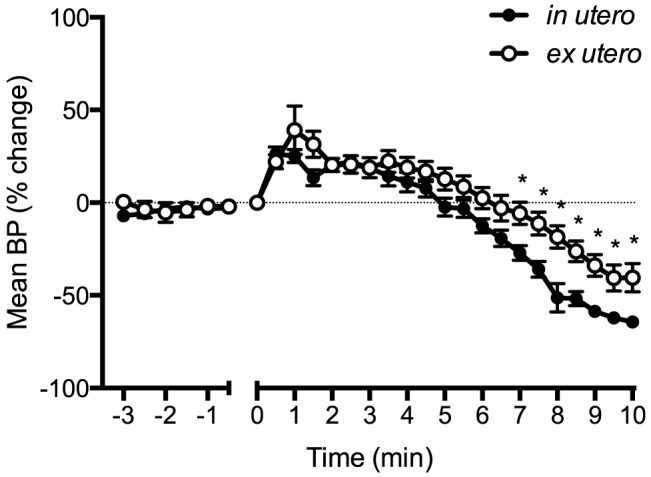
Mean blood pressure (BP), expressed as a percentage change from before cord clamping measured in *in utero* (•) or *ex utero* (○) lambs before and after umbilical cord occlusion (designated as time 0). Data are mean ± SEM. * p<0.05 *in utero* vs *ex utero*.

HR in *ex utero* lambs was significantly higher than *in utero* lambs for the first 10 min of asphyxia. Almost immediately following umbilical cord occlusion, HR in *in utero* lambs rapidly decreased, resulting in a ∼50% reduction within 2 min (Figure 1). In contrast, HR in *ex utero* lambs initially increased following cord occlusion (for ∼1.5 min) by ∼15% before they started to decrease; at 2 min they were not different from before cord occlusion. Mean BP initially increased then decreased in both groups. BP was higher in *ex utero* lambs from 6.5 to 10 min after asphyxia onset (Figure 2).

**Figure 3:**
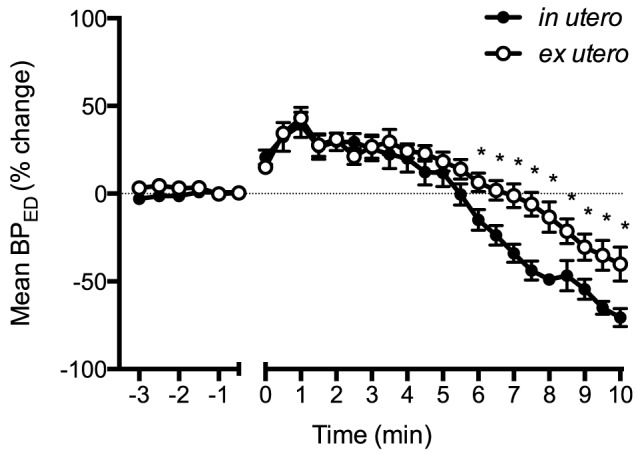
End diastolic pressure (BP_ED_) expressed as a percentage change from before cord clamping measured in *in utero* (•) or *ex utero* (○) lambs before and after umbilical cord occlusion (designated as time 0). Data are mean ± SEM. * p<0.05 *in utero* vs *ex utero*.

**Figure 4:**
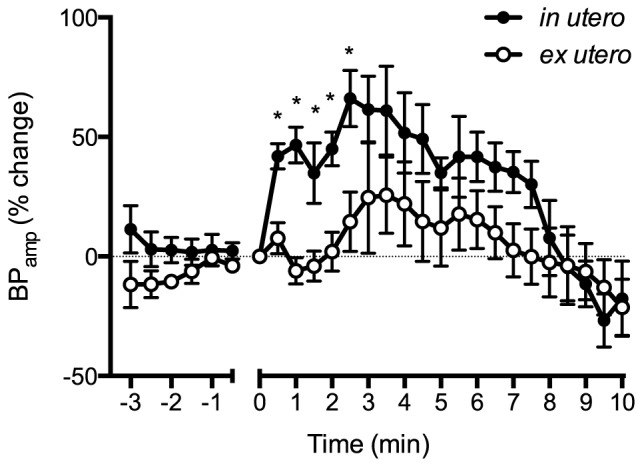
Arterial pressure pulse amplitude (BP_amp_) expressed as a percentage change from before cord clamping measured in *in utero* (•) or *ex utero* (○) lambs before and after umbilical cord occlusion (designated as time 0). Data are mean ± SEM. * p<0.05 *in utero* vs *ex utero*.

**Figure 5:**
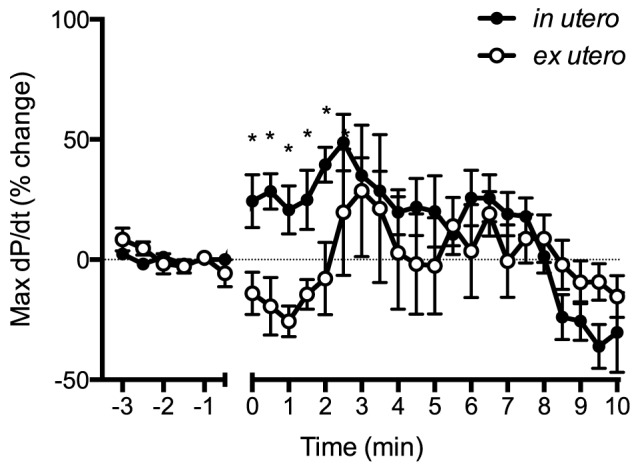
Max dP/dt expressed as a percentage change from before cord clamping measured in *in utero* (•) or *ex utero* (○) lambs before and after umbilical cord occlusion (designated as time 0). Data are mean ± SEM. * p<0.05 *in utero* vs *ex utero*.

BP_ED_ initially increased in both groups by ∼40% within 1.5 min after asphyxia onset in both groups before decreasing (Figure 3). After 6 min of asphyxia, BP_ED_ in *in utero* lambs decreased below *ex utero* lambs. BP_amp_ in *in utero* lambs increased by ∼70% within 2.5 min following cord occlusion, then decreased to pre-occlusion values by ∼9 min (Figure 4). In contrast, BP_amp_ in *ex utero* lambs only increased by ∼25% within the first 3 min of asphyxia before decreasing. Max dP/dt was higher in *in utero* lambs for the first 2 min of asphyxia compared to *ex utero* lambs (Figure 5), with the max dP/dt immediately increasing by ∼50% within 2.5 min of asphyxia in *in utero* lambs before decreasing before. Max dP/dt initially decreased in *ex utero* lambs by ∼25% before increasing.

## Discussion

These data demonstrate that the heart rate and arterial pressure response to asphyxia, induced by umbilical cord occlusion, differs markedly depending upon whether the lambs were *in utero* and submerged in amniotic fluid or were *ex utero* during the period of asphyxia. When lambs remained *in utero*, heart rates fell abruptly in response to umbilical cord occlusion, decreasing by ∼50% within 2 mins (Figure 1). This HR decrease is a well-characterised response to fetal asphyxia, is vagally mediated and is most pronounced when hypoxemia and acidemia are combined [2], [5]. However, if lambs were delivered and asphyxiated *ex utero*, the heart rate initially increased before slowly decreasing, but only decreased to a maximum of ∼35% of pre-occlusion levels at 10 mins.

We consider that the most likely explanation for these findings is a complicated mix of altered stimuli, leading to a modification of the asphyxia-induced, vagal-mediated, bradycardia in *ex utero* lambs. This could have resulted from greater physical stimulation during delivery in *ex utero* lambs (first 30 secs), but this stimulation lasted for <1 min and so is unlikely to account for the continuing differences in heart rate. Indeed, we have recently shown that cord clamping in *ex utero* lambs that are not physically stimulated fails to elicit this increase in HR [9]. Instead, the HR decreases, albeit at a much slower rate and to a lessor degree than that occurs in response to a vagal-mediated bradycardia, as occurs *in utero*. As such, we consider that the vagally-mediated bradycardia may have been modified by the absence of fluid surrounding the lambs face. If correct, this suggests that the vagally-mediated bradycardia that occurs *in utero* in response to asphyxia includes a component of the “diving reflex”. This reflex is a vasovagal response triggered by water contact on the face, resulting in an immediate apnea, bradycardia and peripheral vasoconstriction that is independent of arterial oxygen tension [10], [11]. It is interesting that the original studies described by Dawes and colleagues used fetal monkeys that had water-filled bags placed over their heads to prevent ventilation onset [4]–[7].

It is also interesting that despite major differences in heart rate, the blood pressure was similar between groups, particularly over the first couple of minutes of asphyxia (Figure 2). While this could be partially explained by a greater peripheral vasoconstriction in *in utero* lambs we found that BP_ED_, an indication of downstream vascular resistance, initially increased similarly in both groups (Figure 3). To compensate for the lower HR, *in utero* lambs may have increased combined ventricular stroke volume. This is supported by the finding of a greater increase in BP_amp_ in response to asphyxia, compared to *ex utero* lambs (Figure 4), possibly resulting from increased ventricular contractility. Indeed, max dP/dt, an approximation of heart contractility, was immediately increased in *in utero* lambs upon the onset of asphyxia (Figure 5). However, as preload is reduced following cord clamping, due to the loss in umbilical venous return, this increase in contractility could only have occurred in response to a large increase in sympathetic drive. Indeed, a reduction in preload would normally result in a reduction in stroke volume and likely explains the reduction in max dP/dt we observed in *ex utero* lambs immediately following cord clamping. This suggests that the primary difference in cardiac function response between the two groups of lambs is the change in autonomic drive to the heart in response to cord clamping. That is, the change is much greater in *in utero* lambs, compared to *ex utero* lambs, but further studies are required to elucidate the exact mechanisms.

The immediate increase in stroke volume after the onset of asphyxia in *in utero* lambs is curious. It is widely accepted that the neonate's ability to increase stroke volume in response to bradycardia is limited [12] as the fetal heart is usually performing at the top of its cardiac function curve. As such, it is commonly assumed that neonates respond similarly and can only increase cardiac output by increasing heart rate [12]–[14]. This limitation is believed to be due to immaturity of the myocardium and increased myocardial stiffness [15]. Conversely, other studies have found the Frank Starling relationship is effective in the fetal lamb heart and cardiac output also relies on end-diastolic volume and heart contractility [16]–[18]. These mechanisms exist at birth to enhance neonatal ventricular output [17]. However, these studies were conducted in fetuses and newborns days to weeks after birth. As yet, the changes in cardiac function during the transition at birth are not well understood.

Whatever the mechanism, it is clear that the heart rate response to perinatal asphyxia is complex and possibly includes a component of the “diving reflex” when it occurs *in utero*. As a result, heart rates are not solely determined by oxygenation levels and may not be a good indicator of the extent of the asphyxia or of the underlying cardiovascular function. Indeed, in *ex-utero* lambs, the heart rate initially increased which may have been in response to the physical stimuli. Alternatively it may reflect a sympathetic mediated “stress” response to the hypoxia, as previously described in newborns, which is masked by an over-riding vagal response *in utero* [19]–[21].

It is interesting that, at the end of the asphyxia period, PaCO_2_ levels were higher and SaO_2_ levels were lower in *in utero* lambs compared to *ex utero* lambs. As the recorded values are well outside normal ranges, it is possible that the differences are not real and reflect measurement errors, particularly the saturation levels. However, as the arterial pressure decrease was significantly lower in *in utero* lambs, it is possible that they were more compromised, particularly as arterial pressure was lower than in *ex utero* lambs at the end of the asphyxia period.

These data suggest that our current understanding of the cardiovascular responses to a perinatal asphyxic event do not indicate the stage and severity as accurately as previously assumed. In view of our findings, further studies are urgently required to fully characterise the cardiovascular responses to perinatal asphyxia and to understand how vagal reflexes, such as the diving reflex, may impact on these responses.

## Supporting Information

Table S1 **Heart rate (% change from fetal) of individual** ***in utero*** **and** ***ex utero*** **asphyxia animals from start of asphyxia.**
(PDF)

Table S2 **Mean blood pressure (% change from fetal) of individual** ***in utero*** **and** ***ex utero*** **asphyxia animals from start of asphyxia.**
(PDF)

Table S3 **End diastolic pressure (% change from fetal) of individual** ***in utero*** **and** ***ex utero*** **asphyxia animals from start of asphyxia.**
(PDF)

Table S4 **Pulse amplitude (% change from fetal) of individual** ***in utero*** **and** ***ex utero*** **asphyxia animals from start of asphyxia.**
(PDF)

Table S5 **Max dP/dt (% change from fetal) of individual** ***in utero*** **and** ***ex utero*** **asphyxia animals from start of asphyxia.**
(PDF)
