# Supplementary material for: Circulatory Responses to Asphyxia Differ if the Asphyxia Occurs In Utero or Ex Utero in Near-Term Lambs
Source: PLoS One. 2014 Nov 13;9(11):e112264. doi: 10.1371/journal.pone.0112264 (PMC4230987; doi:10.1371/journal.pone.0112264)
Supplement: Table S1 — Heart rate (% change from fetal) of individual in utero and ex utero asphyxia animals from start of asphyxia. (PDF) [file pone.0112264.s001.pdf]

Table S1. Heart rate (% change from fetal) of individual *in utero* and *ex utero* asphyxia animals from start of asphyxia

	Asphyxia *in utero*										Asphyxia *ex utero*									
time (min)	1	2	3	4	5	6	7	8	mean	SEM	1	2	3	4	5	6	7	8	mean	SEM
fetal	0.0	0.0	0.0	0.0	0.0	0.0	0.0	0.0	0.0	0.0	0.0	0.0	0.0	0.0	0.0	0.0	0.0	0.0	0.0	0.0
0.00	-1.5	-1.0	-0.6	-16.8	1.5	-0.5	-2.1		-3.0	2.3	-1.9	3.2	0.3	-2.1	6.8	0.1	1.1	0.8	1.0	1.0
0.30	-27.9	-15.3	-20.6		-23.7	-5.6	-6.1	-36.0	-19.3	4.2	19.4		6.2	5.3		11.7	27.1	-1.9	11.3	4.3
1.00	-12.4	-7.8	-23.3	-52.4	-34.0	-10.1	-8.5	-21.7	-21.3	5.5	26.1		11.9	10.9	11.7	-1.8	30.8	8.8	14.1	4.2
1.30	-49.1	-28.8	-44.2	-53.4	-46.0	-36.9	-29.5	-36.4	-40.5	3.2	52.9	2.4	17.2	8.0	47.3	-28.7	16.6	-12.2	12.9	9.7
2.00	-49.6	-45.3	-58.9	-52.3	-43.1	-49.7	-37.3	-45.2	-47.7	2.3	20.0	-1.0	6.7	-14.3	77.2	-35.6	-18.2	-20.3	1.8	12.4
2.30	-49.5	-50.3	-59.4	-53.8	-51.6	-58.2	-45.7	-55.0	-52.9	1.6	2.7	-10.0	-22.9	-18.9	30.2	-35.5	-35.1	-20.7	-13.8	7.7
3.00	-49.5	-50.0	-61.6	-56.7	-56.6	-55.8	-48.7	-56.2	-54.4	1.6	-0.4	-54.5	-23.1	-39.0	12.9	-31.8	-38.5	-40.4	-26.9	8.0
3.30	-49.8	-48.3	-59.5	-59.4	-77.5	-50.0	-47.8	-54.0	-55.8	3.5	-11.8	-53.3	-24.4	-43.9	11.9	-32.5	-38.7	-45.3	-29.7	7.5
4.00	-48.8	-46.9	-55.2	-57.9	-74.2	-44.4	-47.5	-48.9	-53.0	3.4	-16.9	-12.2	-26.0	-45.3	20.4	-32.0	-37.9	-47.1	-24.6	7.8
4.30	-46.2	-45.7	-54.5	-56.8	-71.2	-45.7	-44.1	-46.5	-51.3	3.3	-12.1	-14.6	-25.9	-41.9	21.3	-32.6	-34.4	-47.1	-23.4	7.7
5.00	-43.9		-56.7	-57.1	-66.9			-40.1	-52.9	4.9	0.9	-18.9	-24.7	-39.2	17.3	-33.5	-23.6	-46.6	-21.0	7.5
5.30	-43.8	-50.2	-58.5			-47.1	-37.2	-37.6	-45.7	3.3	6.0	-20.7	-46.2	-37.6	10.2	-33.7	-17.7	-45.8	-23.2	7.8
6.00	-47.0	-53.9	-60.1	-57.9	-63.1	-48.0	-38.9	-30.5	-49.9	3.9	-2.1	-22.5	-46.1	-35.2	5.0	-33.4	-24.1	-45.9	-25.5	6.7
6.30	-55.6	-55.1	-59.9	-58.3	-64.3	-49.4	-40.8	-32.7	-52.0	3.7	-12.3	-23.6		-33.5	3.0	-33.4	-26.1	-44.1	-24.3	5.9
7.00	-57.6	-55.0	-60.6	-58.6	-66.2	-50.8	-42.7	-36.8	-53.5	3.4	-21.2	-25.7	-23.2	-34.1	-0.1	-34.2	-26.8	-43.7	-26.1	4.5
7.30	-60.9	-55.8	-61.3	-59.2	-68.3	-52.5	-45.3	-39.7	-55.4	3.3	-20.7	-28.2	-26.5	-35.2	-5.1	-37.1	-30.2	-43.5	-28.3	4.1
8.00	-63.6	-57.3		-59.3					-60.1	1.9	-21.9	-31.0	-28.4	-37.1	-9.2	-38.9	-30.4	-42.7	-30.0	3.8
8.30	-63.3	-59.3	-61.7	-59.9	-50.3	-55.9			-58.4	1.9	-23.5	-33.1	-30.4	-39.4	-12.7	-41.0	-37.3	-42.1	-32.4	3.6
9.00	-63.2	-60.3	-61.4	-60.2	-58.1	-57.6	-52.6	-45.4	-57.3	2.0	-26.3	-36.3	-31.8	-41.6	-16.2	-7.8	-40.4	-42.8	-30.4	4.5
9.30	-60.0		-61.3	-60.6	-61.4	-58.6	-47.8	-47.1	-56.7	2.4	-29.5	-39.4	-34.4	-43.8	-18.4		-43.6	-44.6	-36.2	3.6
10.00	-57.6	-64.0	-61.6	-60.5				-48.5	-58.4	2.7	-32.7	-41.5	-36.7	-46.0	-20.4			-47.5	-37.5	4.1

SEM; standard error of the mean
