# Supplementary material for: Circulatory Responses to Asphyxia Differ if the Asphyxia Occurs In Utero or Ex Utero in Near-Term Lambs
Source: PLoS One. 2014 Nov 13;9(11):e112264. doi: 10.1371/journal.pone.0112264 (PMC4230987; doi:10.1371/journal.pone.0112264)
Supplement: Table S2 — Mean blood pressure (% change from fetal) of individual in utero and ex utero asphyxia animals from start of asphyxia. (PDF) [file pone.0112264.s002.pdf]

Table S2. Mean blood pressure (% change from fetal) of individual *in utero* and *ex utero* asphyxia animals from start of asphyxia

	Asphyxia *in utero*										Asphyxia *ex utero*									
time (min)	1	2	3	4	5	6	7	8	mean	SEM	1	2	3	4	5	6	7	8	mean	SEM
fetal	0.0	0.0	0.0	0.0	0.0	0.0	0.0	0.0	0.0	0.0	0.0	0.0	0.0	0.0	0.0	0.0	0.0	0.0	0.0	0.0
0.00	0.1	20.0	20.7	28.2	18.6	18.6	20.0		18.0	3.0	13.7	11.0	0.8	14.7	14.4	-14.4	-2.2	-8.5	3.7	4.0
0.30	24.3	43.0	30.6		11.4	27.1	28.7	18.4	26.2	3.8	33.7			14.2		14.8	28.2	19.9	22.2	3.8
1.00	9.9	30.5	40.2	13.1	27.9	30.7	28.5	20.8	25.2	3.5	59.8		20.6	112.6	12.6	5.1	36.2	27.4	39.2	13.1
1.30	2.9	25.1	24.2	16.5	23.5	6.7	-9.4	18.0	13.4	4.4	48.3	30.3	22.5	40.6	11.3	-2.6	56.1	46.0	31.6	7.1
2.00	15.8	35.0	18.4	16.4	15.7	21.9	17.5	22.5	20.4	2.3	41.2	19.8	22.8	17.5	26.2	12.1	15.6	8.1	20.4	3.6
2.30	15.3	36.3	18.7	13.5	20.8	26.8	25.1	20.3	22.1	2.6	34.3	6.8	27.3	16.7	17.3	-1.5	36.8	27.9	20.7	4.7
3.00	7.7	33.0	12.1	14.0	13.7	31.1	17.4	20.9	18.8	3.2	29.9	-0.3	15.7	24.4	12.7	-2.7	40.3	31.2	18.9	5.4
3.30	5.4	28.4	16.4	12.8	-15.5	33.4	13.6	20.6	14.4	5.3	27.4	5.2	17.9	26.7	12.2	0.7	48.7	39.1	22.2	5.8
4.00	1.8	24.5	16.6	6.9	-18.5	30.3	10.5	16.7	11.1	5.3	26.4	5.9	13.9	24.6	12.0	-6.6	42.2	33.0	18.9	5.5
4.30	-2.8	18.1	13.6	-1.1	-15.3	26.8	6.6	15.6	7.7	4.8	26.7	3.3	11.8	28.2	8.4	-8.6	36.6	27.8	16.8	5.5
5.00	-11.1		7.0	-4.5	-14.1			10.8	-2.4	4.9	16.3	-4.9	10.9	29.7	3.8	-13.3	33.9	25.2	12.7	5.9
5.30	-17.7	-3.9	3.6			10.4	-16.2	4.9	-3.2	4.8	10.0	-8.0	5.6	30.7	0.2	-16.5	27.6	19.3	8.6	5.9
6.00	-25.8	-9.6	-4.4	-17.0	-22.0	3.8	-21.9	-3.0	-12.5	3.8	5.5	-13.4	1.7	27.9	-3.6	-23.2	16.6	9.0	2.5	5.7
6.30	-40.1	-13.2	-7.9	-25.7	-24.5	-5.1	-28.1	-9.1	-19.2	4.3	-13.3	-17.2		24.5	-6.7	-26.9	13.1	5.8	-3.0	6.9
7.00	-45.7	-26.2	-13.4	-34.7	-30.9	-14.7	-33.8	-17.8	-27.1	4.0	-11.7	-22.3	0.8	21.3	-11.4	-31.5	8.2	1.2	-5.7	6.0
7.30	-56.7	-33.0	-19.2	-42.0	-37.3	-24.2	-44.7	-30.2	-35.9	4.2	-15.6	-27.6	-3.3	17.9	-17.6	-38.2	0.4	-6.1	-11.3	6.2
8.00	-66.5	-41.2		-46.3					-51.3	7.7	-22.4	-34.0	-9.4	12.1	-23.7	-43.6	-10.6	-16.4	-18.5	6.0
8.30	-66.6	-51.9	-37.3	-55.8	-49.9	-48.5			-51.7	3.9	-26.9	-40.1	-13.1	5.7	-29.4	-45.3	-27.8	-32.5	-26.2	5.7
9.00	-69.9	-57.7	-41.8	-59.2	-55.4	-57.6	-71.6	-56.0	-58.7	3.3	-35.8	-49.5	-16.6	-1.1	-34.5	-43.9	-42.6	-46.3	-33.8	5.9
9.30	-71.3		-47.6	-62.0	-61.0	-61.5	-71.4	-59.7	-62.1	3.0	-42.9	-57.0	-22.9	-9.1	-38.5		-55.4	-58.3	-40.6	7.1
10.00	-71.6	-69.9	-52.5	-63.7				-63.8	-64.3	3.3	-50.4	-61.5	-31.0	-17.7	-41.5				-40.4	7.6

SEM; standard error of the mean
