# Supplementary material for: Circulatory Responses to Asphyxia Differ if the Asphyxia Occurs In Utero or Ex Utero in Near-Term Lambs
Source: PLoS One. 2014 Nov 13;9(11):e112264. doi: 10.1371/journal.pone.0112264 (PMC4230987; doi:10.1371/journal.pone.0112264)
Supplement: Table S3 — End diastolic pressure (% change from fetal) of individual in utero and ex utero asphyxia animals from start of asphyxia. (PDF) [file pone.0112264.s003.pdf]

Table S3. End diastolic pressure (% change from fetal) of individual *in utero* and *ex utero* asphyxia animals from start of asphyxia

	Asphyxia *in utero*										Asphyxia *ex utero*								
time (min)	1	2	3	4	5	6	7	8	mean	SEM	1	2	3	4	5	6	7	mean	SEM
fetal	0.0	0.0	0.0	0.0	0.0	0.0	0.0	0.0	0.0	0.0	0.0	0.0	0.0	0.0	0.0	0.0	0.0	0.0	0.0
0.00	-1.9	37.4	22.3	31.1	20.4	18.5	24.1	14.1	20.8	4.1	14.5	25.0	22.8		11.7	2.5	14.5	15.1	3.1
0.30	51.5	60.9	47.7	-6.7	13.4	30.0	32.5	29.9	32.4	8.2	35.3		30.4			37.6		34.4	1.6
1.00	71.5	55.5	57.7	13.7	25.6	34.8	35.6	19.7	39.3	7.2			50.8			47.6	30.7	43.1	6.2
1.30	50.1	53.8	37.8	11.7	26.4	14.3	-3.5	22.1	26.6	7.0		45.1	39.4	31.7	14.3		8.2	27.8	6.5
2.00	41.3	58.2	30.2	17.2	18.2	29.6	23.5	18.6	29.6	5.0	40.8	28.3	37.3	19.9	28.9			31.1	3.4
2.30	45.6	54.2	28.1	16.9	15.9	26.6	28.4	20.3	29.5	4.8		17.0		16.9	14.6	36.5		21.2	4.6
3.00	48.7	55.7	22.7	11.3	-3.7	34.0	16.6	19.5	25.6	6.9	20.1		39.0		6.6	41.3		26.8	6.7
3.30	34.5	52.6	29.1	12.8	-23.4	40.1	14.7	19.1	22.4	8.1	29.0		34.0		7.8	46.7		29.4	7.3
4.00	32.3	45.2	30.9	6.7	-21.4	40.8	10.6	15.3	20.1	7.7	27.6	18.6	29.3	19.4	9.9	41.4		24.4	4.1
4.30	23.2		28.2	-3.7	-17.9	36.7	7.0		12.2	7.4	27.8	13.8	29.0	23.4	5.6	38.2		23.0	4.4
5.00			18.2	-9.7		32.0		7.2	11.9	7.9	16.7	3.8	27.7	25.8	-0.6	36.5		18.3	5.5
5.30	8.5		10.5		-19.8	15.6	-17.5	-0.3	-0.5	6.1	11.1	0.3	18.4	26.9	-5.1	32.1		13.9	5.5
6.00	-6.2	-37.8	4.0	-27.6	-29.5	7.9	-22.2	-7.9	-14.9	5.9	6.3	-7.9	3.0	25.0	-8.6	20.6		6.4	5.3
6.30	-19.6	-48.3	-4.1	-32.2	-32.7	-3.0	-34.7	-15.3	-23.7	5.6	3.5	-14.9	-1.1	22.9	-13.0	14.1		1.9	5.6
7.00	-34.1	-57.2	-11.1	-41.4	-38.8	-16.2	-43.3	-30.4	-34.1	5.3	-15.4	-19.0	15.8	19.7	-18.1	9.6		-1.2	6.9
7.30	-43.8	-66.3	-17.8	-56.3	-47.1	-28.0	-52.5	-38.3	-43.8	5.5	-13.5	-26.0	10.5	15.9	-24.1	1.3		-6.0	6.7
8.00	-50.9				-47.0				-49.0	1.6		-37.8	3.7	10.7	-32.3	-10.7		-13.3	8.7
8.30	-61.6				-49.6	-51.6		-61.3	-56.0	2.6	-30.4	-41.1	-2.0	5.1	-35.8	-24.1		-21.4	7.1
9.00	-58.8	-21.7	-41.2	-66.2	-53.7	-56.9	-72.3	-65.2	-54.5	5.7	-41.3	-47.9	-6.9	-3.3	-40.4	-42.9		-30.5	7.5
9.30	-63.1		-50.0	-76.4	-58.2	-61.9	-76.9	-69.2	-65.1	3.7	-50.0	-53.5	-13.1	-11.3		-48.2		-35.2	8.6
10.00	-63.9	-80.8	-54.4	-81.6				-72.3	-70.6	5.2	-58.6	-59.6	-20.7	-22.0				-40.2	9.8

SEM, standard error of the mean
