# Supplementary material for: Circulatory Responses to Asphyxia Differ if the Asphyxia Occurs In Utero or Ex Utero in Near-Term Lambs
Source: PLoS One. 2014 Nov 13;9(11):e112264. doi: 10.1371/journal.pone.0112264 (PMC4230987; doi:10.1371/journal.pone.0112264)
Supplement: Table S4 — Pulse amplitude (% change from fetal) of individual in utero and ex utero asphyxia animals from start of asphyxia. (PDF) [file pone.0112264.s004.pdf]

Table S4. Pulse amplitude (% change from fetal) of individual *in utero* and *ex utero* asphyxia animals from start of asphyxia

| | Asphyxia *in utero* | | | | | | | | | | Asphyxia *ex utero* | | | | | | | | |
|---|---|---|---|---|---|---|---|---|---|---|---|---|---|---|---|---|---|---|---|
| time (min) | 1 | 2 | 3 | 4 | 5 | 6 | 7 | 8 | mean | SEM | 1 | 2 | 3 | 4 | 5 | 6 | 7 | mean | SEM |
| fetal | 0.0 | 0.0 | 0.0 | 0.0 | 0.0 | 0.0 | 0.0 | 0.0 | 0.0 | 0.0 | 0.0 | 0.0 | 0.0 | 0.0 | 0.0 | 0.0 | 0.0 | 0.0 | 0.0 |
| 0.00 | -0.3 | 4.8 | 42.9 | 58.0 | 36.1 | 31.9 | 15.4 | 7.7 | 24.6 | 7.3 | 15.8 | -21.3 | -4.3 | | -2.5 | -1.4 | -10.1 | -4.0 | 5.0 |
| 0.30 | 37.8 | 63.3 | 46.2 | 33.6 | 33.0 | 39.8 | 22.1 | 59.4 | 41.9 | 5.2 | 21.2 | | 10.8 | | 8.8 | -10.2 | | 7.7 | 6.5 |
| 1.00 | 60.1 | 7.4 | 58.2 | 56.4 | 70.6 | 39.5 | 24.7 | 56.6 | 46.7 | 7.5 | | | -4.0 | | | 2.4 | -16.5 | -6.0 | 5.5 |
| 1.30 | 9.6 | 9.0 | 44.5 | 56.6 | 92.0 | 12.5 | -12.5 | 67.5 | 34.9 | 12.6 | | -7.2 | -8.7 | 22.0 | -18.4 | 5.7 | -17.4 | -4.0 | 6.3 |
| 2.00 | 30.5 | 38.3 | 50.3 | 28.2 | 77.2 | 56.9 | 17.4 | 61.0 | 45.0 | 7.0 | 33.5 | -13.2 | -5.4 | -3.5 | -1.2 | | | 2.0 | 8.1 |
| 2.30 | 54.9 | 59.2 | 69.1 | 23.0 | 125.6 | 103.9 | 45.8 | 47.2 | 66.1 | 11.8 | | -1.4 | | 6.0 | 39.2 | | | 14.6 | 12.5 |
| 3.00 | 33.6 | 41.3 | 44.1 | 51.4 | 147.7 | 91.9 | 49.0 | 33.0 | 61.5 | 14.0 | 50.7 | | -22.0 | | 45.1 | | | 24.6 | 23.4 |
| 3.30 | 42.4 | 37.3 | 37.5 | 46.5 | 189.4 | 61.9 | 41.1 | 32.1 | 61.0 | 18.6 | 29.8 | | -20.3 | | 42.0 | 51.2 | | 25.7 | 15.9 |
| 4.00 | 30.6 | 38.6 | 20.9 | 38.5 | 151.3 | 41.8 | 40.5 | | 51.7 | 16.8 | 13.9 | -15.4 | -23.8 | 96.6 | 31.3 | 29.7 | | 22.0 | 17.6 |
| 4.30 | 37.2 | | 13.4 | 33.9 | 127.9 | 35.2 | 29.7 | 66.9 | 49.2 | 14.4 | 6.4 | -10.2 | -27.1 | 91.7 | 17.1 | 10.0 | | 14.7 | 16.7 |
| 5.00 | | | 20.6 | 32.2 | | 36.3 | | 51.0 | 35.0 | 6.3 | -6.4 | -1.5 | -25.7 | 86.5 | 16.4 | 2.3 | | 11.9 | 15.9 |
| 5.30 | -5.3 | | 28.2 | | 119.2 | 28.4 | 34.1 | 45.3 | 41.7 | 17.0 | -29.0 | -2.3 | 26.8 | 81.7 | 19.9 | 9.3 | | 17.8 | 15.1 |
| 6.00 | 10.8 | 48.8 | 31.3 | 35.4 | 109.2 | 26.9 | 29.6 | 42.1 | 41.8 | 10.4 | -28.2 | 3.2 | 22.3 | 62.4 | 23.3 | 9.6 | | 15.4 | 12.1 |
| 6.30 | 6.6 | 49.9 | 30.7 | 29.2 | 102.3 | 28.9 | 24.0 | 28.1 | 37.5 | 10.2 | -29.5 | 1.8 | 17.5 | 49.2 | 23.8 | -3.0 | | 10.0 | 10.9 |
| 7.00 | 6.3 | 44.3 | 32.9 | 22.8 | 89.1 | 29.9 | 22.6 | 34.3 | 35.3 | 8.6 | 3.5 | -4.5 | -36.8 | 42.8 | 20.4 | -10.2 | | 2.5 | 11.1 |
| 7.30 | -10.5 | 53.5 | 35.1 | 25.2 | 67.3 | 25.8 | 14.7 | | 30.2 | 9.6 | 8.0 | 0.1 | -38.8 | 39.6 | 16.1 | -25.1 | | 0.0 | 11.6 |
| 8.00 | -25.5 | 42.0 | | | -11.2 | | | 25.6 | 7.7 | 15.7 | | 15.2 | -39.2 | 36.8 | 7.0 | -32.4 | | -2.5 | 14.5 |
| 8.30 | -43.6 | 33.4 | | | -17.6 | -14.6 | | 18.6 | -4.8 | 13.7 | 43.1 | -2.3 | -38.5 | 31.6 | 4.0 | -60.5 | | -3.8 | 16.3 |
| 9.00 | -53.2 | 15.1 | 18.4 | 5.2 | -26.6 | -26.4 | -35.0 | 11.0 | -11.4 | 9.6 | | -11.7 | -38.8 | 24.5 | 0.6 | | | -6.4 | 11.8 |
| 9.30 | -58.4 | | -4.2 | 11.6 | -50.5 | -40.4 | -48.9 | 3.6 | -26.7 | 11.1 | | -23.7 | -41.9 | 18.3 | -4.6 | | | -13.0 | 11.6 |
| 10.00 | -60.0 | -10.5 | -15.7 | 15.6 | | | | | -17.7 | 15.7 | | -41.0 | -45.4 | 9.1 | -7.7 | | | -21.2 | 11.8 |

SEM, standard error of the mean
