# Supplementary material for: Circulatory Responses to Asphyxia Differ if the Asphyxia Occurs In Utero or Ex Utero in Near-Term Lambs
Source: PLoS One. 2014 Nov 13;9(11):e112264. doi: 10.1371/journal.pone.0112264 (PMC4230987; doi:10.1371/journal.pone.0112264)
Supplement: Table S5 — Max dP/dt (% change from fetal) of individual in utero and ex utero asphyxia animals from start of asphyxia. (PDF) [file pone.0112264.s005.pdf]

Table S5. Max dP/dt (% change from fetal) of individual *in utero* and *ex utero* asphyxia animals from start of asphyxia

	Asphyxia *in utero*										Asphyxia *ex utero*								
time (min)	1	2	3	4	5	6	7	8	mean	SEM	1	2	3	4	5	6	7	mean	SEM
fetal	0.0	0.0	0.0	0.0	0.0	0.0	0.0	0.0	0.0	0.0	0.0	0.0	0.0	0.0	0.0	0.0	0.0	0.0	0.0
0.00	29.3	-8.9	46.2	83.9	18.9	21.4	19.3	-15.4	24.4	11.0	-56.2	-16.3	-7.4	0.3	0.6	-13.6	-5.5	-14.0	8.8
0.30	45.4	59.4	38.4		13.3	20.8	14.0	7.6	28.4	7.4	-51.3	-19.4		-21.0			14.1	-19.4	12.0
1.00	0.6	-17.1	48.3	57.0	47.8	16.9	22.3	-10.0	20.7	10.0	-33.5		-3.1	-44.7	-31.4	-21.8	-19.6	-25.7	6.4
1.30	11.6	-1.0	24.9	68.6	79.6	-6.8	-14.6	36.8	24.9	12.3		7.3	-9.0	-23.1	-23.1		-24.3	-14.5	6.1
2.00	30.2	46.8	21.1	35.4	74.6	33.0	13.5	61.6	39.5	7.2	-61.7	-4.8	-6.6	-13.0	-12.6	-11.0	54.3	-7.9	15.1
2.30	13.9	38.2	12.9	36.1	113.8	66.4	42.0	66.7	48.7	11.7	-65.2	18.7		-14.5	9.0	76.0	94.4	19.7	26.3
3.00	14.1	26.5	10.2	66.0		46.0	41.9	40.8	35.0	7.4	-57.5	55.7	-12.2		2.3	79.8	103.7	28.6	27.4
3.30	4.7	19.2		63.5		22.2	36.8	25.2	28.6	8.2	-63.9		-14.1		2.7	76.5	105.3	21.3	30.8
4.00	8.8		-11.1	54.2		12.6	38.0	15.7	19.7	9.4	-68.4	-17.4	-15.7	-12.3	-8.6	48.4	93.3	2.8	23.4
4.30			-17.5	44.6		9.3	26.3	46.7	21.9	12.0	-72.1	-13.5	-17.2	-12.0	-10.8	36.2	76.8	-1.8	20.9
5.00			-9.2	39.1				30.2	20.0	14.9	-69.7	-15.1	-14.7		-7.1	29.4	61.6	-2.6	20.0
5.30		16.9	-3.5			3.3	22.7	13.3	10.5	4.7		-4.5		-5.5	0.0	22.8	57.1	14.0	11.9
6.00	-9.2	25.6	2.3	47.4	93.3	1.5	27.7	16.4	25.6	11.6	-71.6	-3.2		4.4	2.9	33.2	55.8	3.6	19.3
6.30	-4.4	23.2	4.8	39.9	85.1	6.9	25.9	23.5	25.6	9.9		12.7		-1.1	2.3	36.1	45.3	19.1	9.2
7.00	-17.3	22.8	4.3	30.1	71.4	5.0	19.0	16.1	18.9	9.1	-59.6	10.2	-26.1	0.6	1.2	28.5	40.9	-0.6	15.1
7.30		19.3	7.7	21.8	52.6	-1.3	7.7		18.0	7.7		18.1	-27.4	4.5	-2.8	22.1	38.1	8.8	10.2
8.00		10.7			-12.1			5.4	1.4	6.9		23.6	-27.7	11.9	-4.6	14.4	34.5	8.7	9.9
8.30	-56.0	4.0			-16.3	-31.9	-40.2	-2.7	-23.8	9.4	-35.8	15.6	-27.7	9.0	-7.5	3.2	28.0	-2.2	10.3
9.00	-60.9	-6.4	-11.2	2.8	-28.3	-40.6	-49.2	-10.8	-25.6	8.1	-19.3	3.3	-31.4	7.1	-14.0	-31.2	19.7	-9.4	8.9
9.30	-62.9		-17.5	1.5	-49.4	-50.4	-55.3	-18.9	-36.1	9.2	-5.1	-17.2	-35.2	3.5	-14.4		12.7	-9.3	7.5
10.00	-61.3		-25.0	-4.4					-30.2	16.6	8.1	-31.8	-42.0	-9.0	-19.3		2.2	-15.3	8.7

Max dP/dt, Maximum rate of blood pressure increase during systole; SEM, standard error of the mean
